# The Diagnosis and Treatment of Tuberculous Infections

**Published:** 1920-12-04

**Authors:** 


### JOINT DISEASE IN CHILDREN.

### The Diagnosis and Treatment of Tuberculous Infections.

THE American quarterly publication* known as "International Clinics" forms a valuable addition to any medical library on this side of the Atlantic, and although the majority of contributions to its pages originate in the United States, yet there are many valuable records included of advances made in our own country. In the following note we make particular mention of a post-graduate lecture likely to interest many institutional readers, and given by the Associate Professor in Surgery to the University of Pennsylvania. It forms a good example of the clarity of style and thought with which the highly interesting pages of these volumes are compiled.

In basing our few remarks concerning this condition upon Dr. Ashhurst's words, it is unnecessary to detail the accepted modes of entry of the tubercle bacilli to the young organism. Reaching the cervical, bronchial, or mesenteric lymph glands the organisms ultimately find their way into the circulation, and if a suitable soil is found, a *locus minoris resistentiæ*, then multiplication and the typical lesions of tuberculosis are produced. It is suggested that the most favourable sites for this process to occur are those where lymphoid cells exist, and, in young children, the synovial lining of joints, the red bone marrow at points such as the cancellous ends of bones, and in the short bones of the hand and foot, form the most likely and vulnerable spots. Even in these positions, if the local resistance is sufficient, no lesion may develop, but if the natural barriers to infection are for any reason lowered, the result is obvious. Thus it is that one finds, in about one-third of the cases of tuberculous joints, a history of trauma, usually comparatively slight, preceding, by a period of several weeks, the development of symptoms of tuberculous disease. In view of the frequency with which children sustain slight joint strains, etc., it seems reasonable to assume that, even if it cannot be demonstrated clinically, the child with a tuberculous bone or joint disease has a tuberculous focus somewhere else in its body.

The author makes a great point of the importance of referred pain. For example, there is, he says, scarcely a year passes that one does not see one or more little children who have been treated symptomatically by their family doctors for weeks, sometimes for months, for pains in their stomachs, when a cursory examination of their back shows them to be suffering from tuberculosis of the spine, and demonstrates that the pain is really referred along the intercostal or lumbar nerves. So, also, with hip-joint disease, the pain may be referred to the knee along the obturator nerve. On the other hand, stiffness of a joint may be noticed at once by parents, it may wear off during the early part of the day and again become pronounced in the evening. Sometimes a joint, painless by day, may produce "starting pains" at night when the muscles are relaxed, but it is in relatively few cases that attention is at once specifically drawn to the exact site of the infection.

* "International Clinics." Lippincott & Co., Philadelphia and London. Price £2 2s. per set of 4 volumes.

### PHYSICAL EXAMINATION.

For this reason, then, it is impossible to insist too strongly that children should always be examined stripped. The presence of clothing inevitably hinders the physician's or nurse's perception of deformity or limitation of movement. Also it is essential to gain the child's confidence in order to watch it carrying out simple unobstructed and unassisted movements. The child should, for instance, stoop to pick up something off the floor; if there is spinal disease one may often see him bend his knees and hips, bringing his buttocks almost into contact with the floor rather than flex his spine to stoop forward and pick up the object normally. The spasm of the associated muscles must also be noted and the limitation of movement thereby produced. It is an axiom insistently put to the medical student, but nevertheless forgotten with ridiculous frequency, that *the* only satisfactory way to observe movement limitation is to compare the diseased with the corresponding healthy joint. Details of comparisons which might then be drawn would take us into many pages. Dr. Ashhurst gives the fullest description of a list of interesting points, all amply illustrated by photographs taken at his clinic. The immediate lesson, however, is that the greatest care is essential in observing the early manifestations. Once any considerable degree of involvement of bone and cartilage or its destruction has occurred the diagnosis is usually obvious at a glance, but if satisfactory care is to be exercised the condition positively must be detected early, and to do this requires remarkably close observation of the child and its everyday movements.

### TREATMENT.

And as to cure, the author insists that the following still holds good. Local treatment may be summed up in one word—Rest. Yet it is a curious thing that we do not, strictly speaking, know the *modus operandi* of rest in this capacity. A plausible theory has been suggested on the lines that the "cure" is effected by abolishing all joint friction, because thus both red marrow and synovia become atrophic and even disappear entirely in the event of ankylosis. Where these tissues do not exist it is supposed that tubercle bacilli cannot live, but whatever the true explanation, the facts remain that, apart of course from general constitutional and hygienic treatment, our best methods obtain rest by a combination of fixation and traction of the parts involved. Here again it is obvious that to describe the technical application of this technique would take us into a mass of detailed orthopædics, and it must suffice to remember the underlying principle of all of it.

Treatment of Cold Abscess.

There is another aspect to remember. It does occasionally happen that under simple rest treatment a bone lesion which has gone on to pus formation may clear up. But more frequently it approaches nearer and nearer to the skin, with consequent danger of rupture and secondary infection with pus-forming skin organisms. At this point the author urges that an opening, under a local anæsthetic, should be made with the strictest asepsis, the cavity cleaned out with iodine and the incision made then sutured in layers without drainage. "It is dangerous to leave a cold abscess alone until the overlying skin has become adherent and reddened, since secondary infection is very frequent and rapid joint disintegration, hectic, amyloid disease, etc., follow; and it is still more dangerous to open a cold abscess without a perfectly aseptic technique, or to drain it by tube or gauze after incision, or to allow it to discharge itself spontaneously. Aspiration of a cold abscess . . . is inferior to formal incision, because it cannot be done satisfactorily until pus is very close to the surface and unless it is very fluid."

Operative treatment of the surgical deformities following tuberculous infection are beyond the scope of this note, but it is well to remember that there is always a possibility of lighting up the old infection once more, and we have always to decide whether the patient's disability is so pronounced as to demand surgical relief. Ankylosis is probably the best and surest "cure" of tuberculous arthritis, and, except in the elbow or shoulder, is usually best not interfered with.

It is obvious, therefore, that the treatment of this infection, when it has begun, is inevitably a long and tedious process, and the extreme urgency of *early* diagnosis lays a duty on nurses and doctors alike when dealing with juvenile cases. If proper facilities are not at hand transference of their patients to the advantages of a hospital service is essential, for there only can the obvious necessity of resisting the beginning of the pathological process be efficiently met.

